# Magnetic Interactions in Wiegand Wires Evaluated by First-Order Reversal Curves

**DOI:** 10.3390/ma15175936

**Published:** 2022-08-27

**Authors:** Guorong Sha, Chao Yang, Zenglu Song, Yasushi Takemura

**Affiliations:** 1 Department of Electrical and Computer Engineering, Yokohama National University, Yokohama 240-8501, Japan; 2 School of Transportation Engineering, Nanjing Vocational University of Industry Technology, Nanjing 210023, China

**Keywords:** Wiegand wire, FeCoV wire, magnetization reversal, first-order reversal curve (FORC), large Barkhausen jump

## Abstract

Wiegand wires exhibit a unique fast magnetization reversal feature in the soft layer that is accompanied by a large Barkhausen jump, which is also known as the Wiegand effect. However, the magnetic structure and interaction in Wiegand wires cannot be evaluated by conventional magnetization hysteresis curves. We analyzed the magnetic properties of Wiegand wires at various lengths by measuring the first-order reversal curves (FORCs) and by evaluating the FORC diagram from a series of FORCs. In particular, we used a FeCoV Wiegand wire with a magnetic soft outer layer, an intermediate layer, and a hard core. The magnetization of the various layers in the wire could be identified from the FORC diagrams. Furthermore, based on the interaction between multiple layers, the positive and negative polarity of the FORC distribution was clarified.

## 1. Introduction

Magnetization reversal in magnetic wires with bistable magnetization states is accompanied by a large Barkhausen jump, known as the Wiegand effect [1]. The Wiegand sensor is a type of magnetic sensor that is manufactured using the Wiegand effect. It can directly convert a magnetic signal into an electrical signal without using any external power supply. The sensor contains a specially processed Vicalloy wire and an induction coil wound around its periphery. This special processed Vicalloy wire is referred to as the Wiegand wire. With a typical composition of Fe_0.4_Co_0.5_V_0.1_, it has been identified as the optimum material for realizing the Wiegand effect [2,3]. The Wiegand wire can be fabricated from a conventional wire of a suitable magnetic material by using a method that principally consists of torsional stress, annealing, and cold treatment to introduce differences in the coercivity of the wire shell and wire core [1].

Typically, the Wiegand wire is composed of a dual magnetic structure with a soft magnetic shell and hard magnetic core. The coercive fields of the soft layer and hard core are approximately μ_0_H_c_ = 2 mT and 8 mT, respectively. Previous studies have reported [2] that the volume ratio of the soft layer can reach values up to 23%, with the thickness of the soft layer at approximately 0.03 mm. Using the magnetic gradient between the interior and exterior of the Wiegand wire, the magnetization direction of the soft layer flips instantaneously when subjected to the excitation of an alternating magnetic field of adequate intensity. The magnetization of the soft layer is aligned to the same or opposite direction to that of the hard core, and the magnetic field around the Wiegand wire varies abruptly to induce a group of positive or negative electrical pulses in the induction coil [4]. If the alignment direction is the same, a demagnetizing field is generated [5], whereby the demagnetization factor mainly involves the length, diameter, and degree of magnetization of the Wiegand wire. In the case of an antiparallel alignment, magnetostatic coupling [6] is generated at both ends of the wire. Consequently, the amplitude of the electrical pulses can attain several volts with widths approximately equal to 10 µs [7]. In particular, the pulse amplitude is related only to the intensity of the excitation magnetic field and is not influenced by the rate of change of the magnetic field [8]. Therefore, the Wiegand sensor does not require an external power supply. In general, the working frequency of the Wiegand sensor ranges from 0 to 20 kHz, and the polarity of the pulse can be determined based on that of the excitation magnetic field [9]. In addition, Wiegand sensors are compatible with digital systems, wherein the system does not contain movable components or semiconductor devices, as they are built to be suitable for use in adverse environments and places that would be unattended for long-term periods. Capitalizing on these advantages, the application of a Wiegand sensor is no longer limited to the generation of an output signal. By contrast, it can be used as a power supply for a variety of low-power devices [10,11]. Thus, researchers are investigating the magnetic characteristics of Wiegand wires to exploit the advantages and application potential of Wiegand sensors, especially as a power supply for certain wireless electronic devices operating in environments without power supply.

To understand the underlying reason for the electric pulse induced in the Wiegand wire, maximize the power generated by the Wiegand sensor, and improve its application ability, the magnetic structure and the internal interaction in the Wiegand wire should be characterized. Although the magnetization curves of both the major and minor loops [2], and the relationships of the magnetization processes of Wiegand wires—including their magnetic structures—have been reported earlier, the details of the magnetic structure and the internal interactions in the Wiegand wire have not yet been completely understood.

We previously analyzed the FORC diagram of a Wiegand wire with a length of 5 mm based on a simple model of the two-layer magnetic structure of the wire [11] and observed no clear boundary between the two layers. The applied magnetic fields (μ_0_H) and reversal fields (μ_0_H_r_) of different regions or their peaks in the FORC diagram were used to compare the switching fields of the minor loops and coercivities of the soft layer and hard core of the Wiegand wire.

The present study aims to elucidate the internal interaction in the Wiegand wire based on the three-layer magnetic structure. Accordingly, we analyzed the magnetization process and the switching field by using minor loops, FORCs, and FORC diagrams of Wiegand wires with various lengths. The coercive field (μ_0_H_c_) and the interaction field (μ_0_H_int_) were analyzed comprehensively according to the evolution law of the FORC distribution. We examined the distribution of negative value in the FORC diagrams and studied the internal interactions in the wires by using the mathematical definition of the FORC distribution.

## 2. Materials and Methods

**Figure 1:**
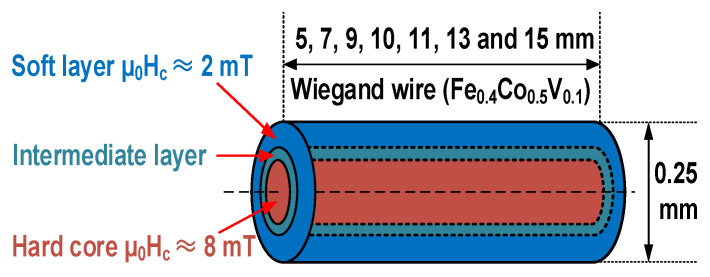
Schematic of magnetic structure and size of Wiegand wire.

In this study, seven Wiegand wires were used which had lengths equal to 5, 7, 9, 10, 11, 13, and 15 mm, and diameters equal to 0.25 mm. When the Wiegand wire is magnetized in the applied magnetic field, a reverse additional magnetic field, i.e., a demagnetizing field is generated. The demagnetizing field is inversely proportional to the length of the Wiegand wire. The different lengths of the Wiegand wire may also affect the bias of the switching field of the Barkhausen jump in the applied magnetic field. The Wiegand wire was made of a single material composed of Fe_0.4_Co_0.5_V_0.1_ (SWFE, Co. Ltd., Meishan, China), and the multilayered magnetic structure was achieved by torsional stress, annealing, and cold treating the wire. The magnetic structure of the Wiegand wire with its soft outer layer and hard core is illustrated in Figure 1. The coercive fields of the soft layer and hard core were approximately µ_0_H_c_ = 2 and 8 mT, respectively. This is a conventional magnetic structure that was used to interpret previously reported results [2,7]. In this study, the coercivity was considered to vary gradually along the radial direction of the wire [11], i.e., no evident boundary exists between the soft layer and hard core. Instead, a transition region is present between the soft layer and hard core, which can be referred to as the intermediate layer.

**Figure 2:**
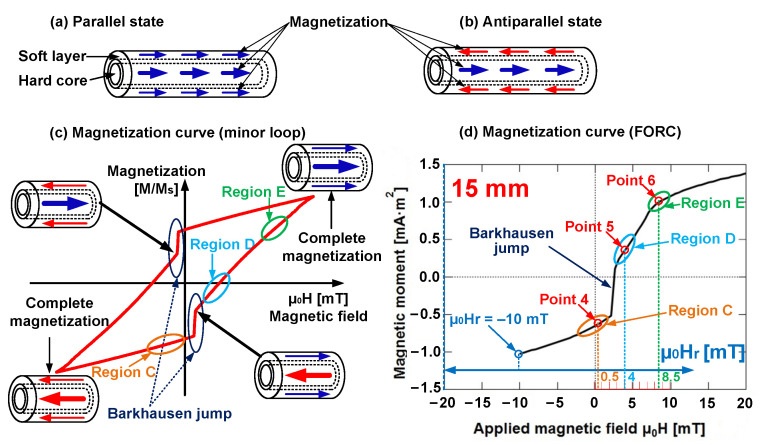
Magnetization states and magnetization curves of Wiegand wire: (**a**) parallel state; (**b**) antiparallel state; (**c**) magnetization curve (minor loop); and (**d**) magnetization curve (one first-order reversal curve (FORC)).

As illustrated in Figure 2a,b, the magnetization alignment of the soft layer and hard core of the Wiegand wire can either exist in parallel or antiparallel states. The magnetization reversal of the soft layer is accompanied by a large Barkhausen jump that is known as the Wiegand effect, as illustrated in Figure 2c,d [12,13]. Figure 2d is a FORC curve with a Wiegand wire with a length of 15 mm when μ_0_H_r_ = −10 mT. The detail information of points and regions in Figure 2c,d as described later s in this study.

Vibrating sample magnetometry (VSM) is one of the crucial methods for measuring the magnetism of materials. In particular, it can measure the basic magnetic properties of magnetic materials such as the magnetization curve, hysteresis loop, demagnetization curve, temperature rise curve, temperature rise/drop curve, temperature drop curve, temperature variation with time, and FORCs. Notably, it can obtain various corresponding magnetic parameters, such as saturation magnetization, residual magnetization, coercivity, magnetic energy product, Curie temperature, and permeability (including initial permeability). VSM is extensively used in the magnetic measurements and research of various ferromagnetic, antiferromagnetic, paramagnetic, and diamagnetic materials [14].

Typically, the magnetic properties of magnetic materials are expressed by measuring the major or minor hysteresis loops. Prior research has already investigated the magnetization reversal process of magnetic materials in detail by the magneto-optical Kerr effect (MOKE) [15,16]. Nonetheless, the information on the interactions or coercivity distributions cannot be feasibly obtained from the major hysteresis loop or the minor loop, as the MOKE can only evaluate the magnetization process on the surface of magnetic materials. If the sample comprises thin films or nanowires of a thin structure, the surface magnetization is similar to the surface layer or the whole sample. Nakamura et al. showed that the surface magnetization of a Wiegand wire measured by MOKE is completely different from that of the whole layer or soft layer near the surface region [15]. Different magnetization at the surface is possible for bulk material, as well as a Wiegand wire of sub millimeter material. In these regards, the FORCs and FORC diagrams can be employed as a superior method to obtain information on interactions, coercivity distributions, the identification of multiple phases, and the relative proportions of reversible and irreversible components of the magnetization of Wiegand wire [17,18,19]. The magnetization curves of the major and minor hysteresis loops along with the FORCs were measured in this study using a vibrating sample magnetometer (model 8600 series, Lake Shore Cryotronics, Westerville, OH, USA) at room temperature.

**Figure 3:**
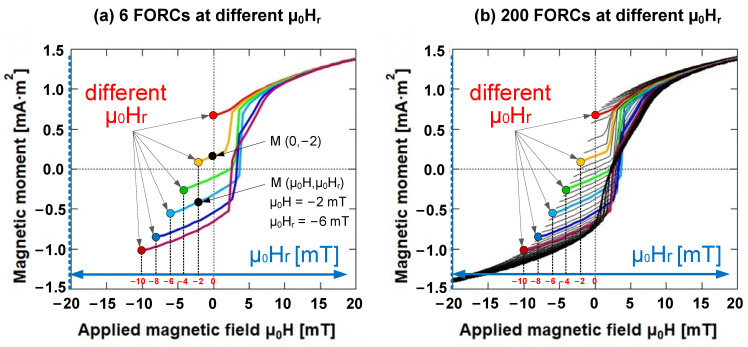
Example of FORCs of Wiegand wire at various μ_0_H_r_. (**a**) 6 FORCs at various μ_0_H_r_. Two black points of magnetization on multiple FORCs at two applied magnetic fields (μ_0_H) with two reversal fields (μ_0_H_r_) are represented by M (μ_0_H = −2 mT, μ_0_H_r_ = −6 mT) and M (μ_0_H = 0 mT, μ_0_H_r_ = −2 mT), respectively; (**b**) 200 FORCs at various μ_0_H_r_.

Specifically, a FORC was measured by saturating a sample in a large positive applied magnetic field (μ_0_H_sat_), which diminished the field to a reversal field (μ_0_H_r_). The FORC represents the magnetization curve obtained when the applied field is swept back to the saturation field (μ_0_H_sat_) in a series of regular field steps (μ_0_H). Upon repeating this measurement for various reversal fields (μ_0_H_r_), a series of FORCs was obtained [20,21]. The magnetization at any applied field (μ_0_H) on the FORC with the reversal field (μ_0_H_r_) can be expressed as M (μ_0_H, μ_0_H_r_), where μ_0_H ≥ μ_0_H_r_ (Figure 3).

A FORC diagram can be obtained from a series of partial hysteresis curves known as FORCs [19]. The FORC distribution *ρ*(*H, H_r_*) is defined as a mixed second derivative, such as,

(1) $$\rho (H,H_{r})=–\frac{1}{2}\frac{\partial^{2}M(H,H_{r})}{\partial H_{r}\partial H}=–\frac{1}{2}\frac{\partial }{\partial H_{r}}\left(\frac{\partial M(H,H_{r})}{\partial H}\right)$$

The resulting plot of *ρ*(*H, H*_r_), known as the FORC distribution, maps the introduction of hysteresis, or irreversible change in moment, from the initial state [22].

To analyze the magnetic properties of the Wiegand wire, the axes were rotated by varying the coordinates from (*H, H*_r_) to (*H*_c_*, H*_int_):

(2) $$H_{c}=\frac{H-H_{r}}{2},$$

(3) $$H_{\mathrm{int}}=\frac{H+H_{r}}{2},$$

In quantitative FORC analyses, the diagrams are projected on the *H*_c_ and *H*_int_ axis by integrating over the *H* and *H*_r_ axes, respectively; the former is referred to as the coercive field distribution, and the latter is referred to as the interaction field distribution [23].

**Figure 4:**
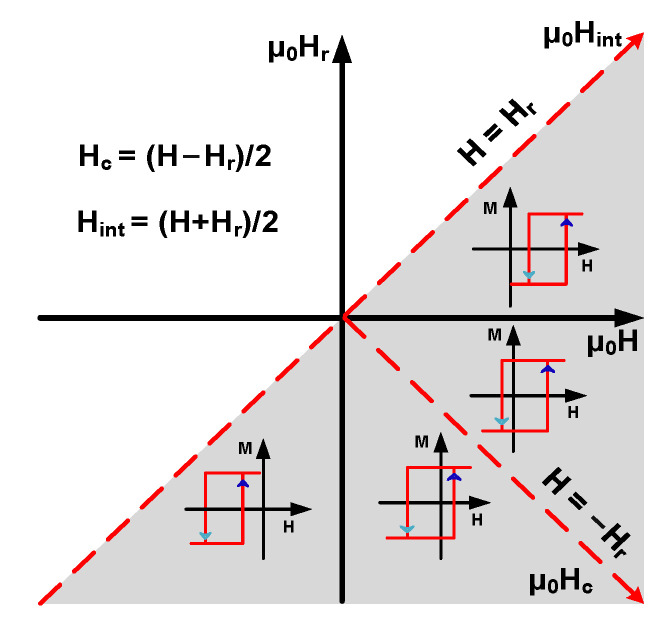
Two-dimensional coordinate system composed of H and H_r_, and coordinate of switching field and interaction field with a rotation angle of 45° composed of H_c_ and H_int_.

In the two-dimensional coordinate system composed of *H* and *H*_r_, the geometric interpretation of function *ρ*(*H*, *H*_r_) reflects the FORC distribution contour plots projected in the gray triangle area (boundary defined by ± μ_0_H_sat_) in Figure 4 [24].

The FORC distribution was derived from the differential component of the gradient of the magnetization curve, and it principally emphasizes the irreversible magnetization of the sample [11]. Accordingly, we can determine the points and regions on the FORC curve that bear one-to-one correspondence with the FORC diagram.

**Figure 5:**
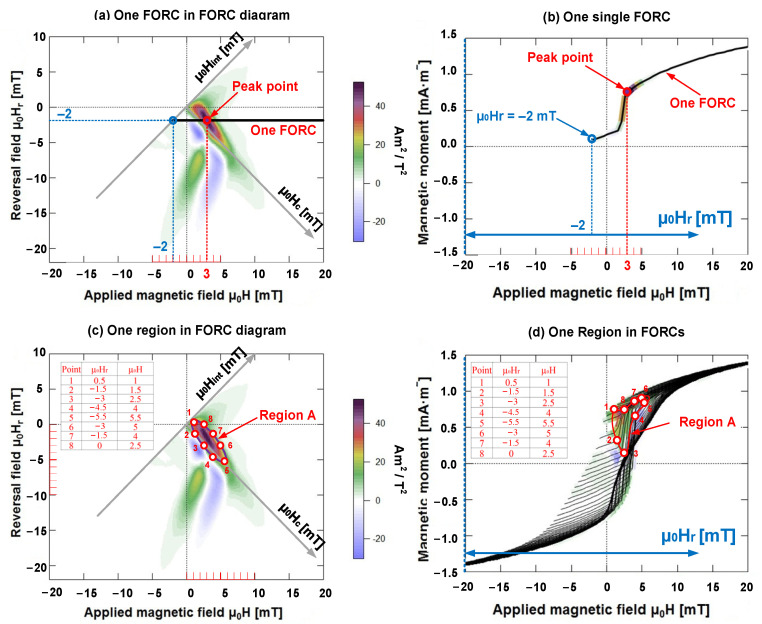
FORC diagram and FORCs. (**a**,**b**) FORC curve corresponds to the horizontal line in FORC diagram. (**c**,**d**) Several FORCs corresponding to a region in the FORC diagram.

As illustrated in Figure 5a,b, a FORC curve corresponds to a horizontal line in the FORC diagram. The blue point (μ_0_H = −2 mT, μ_0_H_r_ = −2 mT) denotes the start point. If the applied magnetic field strength is a saturated magnetic field (μ_0_H = μ_0_H_sat_, μ_0_H_r_ = −2 mT), it indicates the end point of the FORC curve. The purple point plots the peak point (μ_0_H = 3 mT, μ_0_H_r_ = −2 mT), which is the maximum intensity of the FORC distribution.

As illustrated in Figure 5c,d, several FORCs corresponded to a region in the FORC diagram. Let us consider region A as an example. To accurately locate region A on the FORCs, we selected eight important points on the contour of region A in the FORC diagram and calculated the coordinates of their reversal fields μ_0_H_r_ and applied magnetic fields μ_0_H. Thereafter, we determined the coordinates of the reversal fields μ_0_H_r_ and the applied magnetic fields μ_0_H of the eight points on FORCs, and subsequently, connected these eight points with the curves on FORCs. The area surrounded by the closed-curve is region A in the FORC diagram.

## 3. Results

### 3.1. Magnetization Curves of Major and Minor Hysteresis Loops

**Figure 6:**
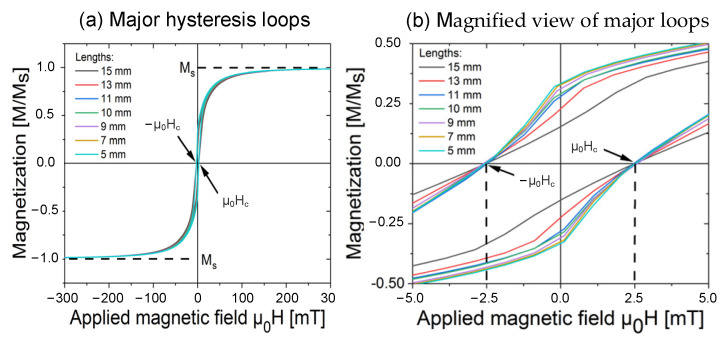
(**a**) Magnetization curves (major loops) of Wiegand wires at varying lengths and at a diameter of 0.25 mm. (**b**) Magnified views of the major loops for the applied field μ_0_H from −5 to 5 mT.

The magnetization curves (major loops) of Wiegand wires at various lengths are illustrated in Figure 6a. In particular, the magnetization was normalized according to their saturation magnetization to compare the magnetization at varying lengths. The magnified view of the major loops for the applied field μ_0_H from −5 to 5 mT is depicted in Figure 6b.

As shown in Figure 6b, the coercivity of the Wiegand wire of the same material was almost constant, approximately equal to 2.5 mT, and it did not vary with the Wiegand wire length. The major hysteresis loops were symmetric magnetization curves of the whole sample. In a major hysteresis loop, the measured coercivity is the weighted coercivity of the entire ensemble of magnetic particles that constitute a magnetic material, thus, if the material contains more than one magnetic phase, it is hard to discern between these phases.

FORCs are a series of asymmetric magnetization curves, and FORCs can provide information that is not possible to obtain from the hysteresis loop alone. This includes the distribution of coercivity and interaction fields and the identification of multiple phases in composite or hybrid materials containing more than one phase [18].

The relationship between the coercivity of the Wiegand wire and its FORC distribution is discussed further in this study.

**Figure 7:**
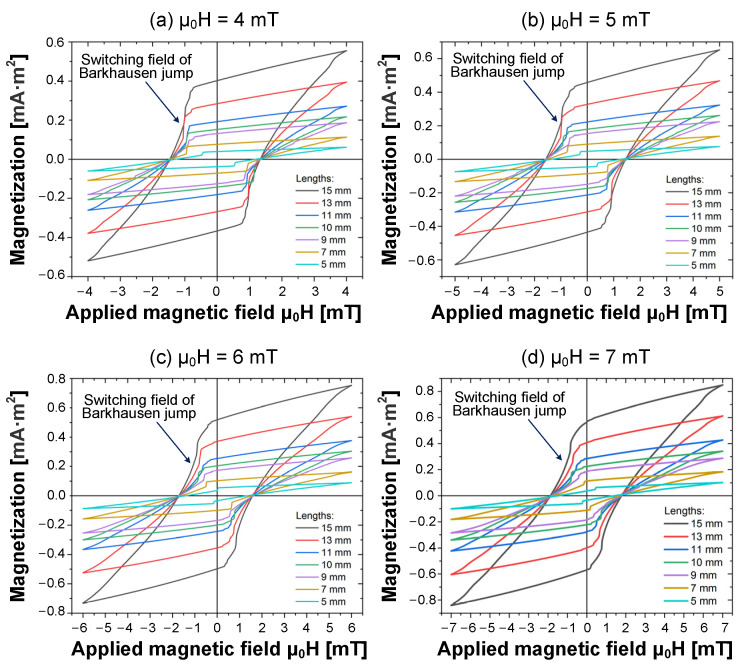
Magnetization curves (minor loops) of Wiegand wires at various lengths and at a diameter of 0.25 mm. Applied magnetic field intensities at (**a**) μ_0_H = 4 mT; (**b**) μ_0_H = 5 mT; (**c**) μ_0_H = 6 mT; and (**d**) μ_0_H = 7 mT.

The minor loops of Wiegand wires with varying lengths were traced using an applied alternating magnetic field of μ_0_H in the range of 2 to 15 mT. Subject to the action of a specific magnetic field intensity on Wiegand wires of varying lengths, Barkhausen jumps were observed through these minor loops, as illustrated in Figure 7.

In these minor loops, at an equal intensity of the applied magnetic field, we observed that the magnetization of the Wiegand wire decreased as a function of the length, and the switching field of the Barkhausen jump decreased as the Wiegand wire length decreased. Even at some intensity of the applied magnetic field, the switching field of the shortest Wiegand wires appears at μ_0_H = 0, as illustrated in Figure 7c, or the polarity of the switching field of the shortest Wiegand wires is opposite to the switching field of other length values, as illustrated in Figure 7d. This was attributed to the fact that the switching field intensity varied with the demagnetizing field. By decreasing the length of Wiegand wire, the demagnetizing field in the wire increases. The soft layer of short-length wires is autonomously reversed in the positive magnetic field range. The critical length of this phenomenon was 7 mm, as shown in Figure 7d. Owing to the asymmetric magnetic structure of the soft layer and hard core, the parallel and antiparallel switching fields in the magnetized state are not equivalent [25].

### 3.2. First-Order Reversal Curves and FORC Diagrams

There are certain open-source software programs such as FORCinel [26] and VARIFORC [27] that can be used to calculate the FORC distributions and plot FORC diagrams. In this study, FORCinel and its auxiliary software (Igor Pro^®^, WaveMetrics Inc., Portland, OR, USA) were used, because the advanced functions of VARIFORC can be selected from the FORCinel menu [11].

**Figure 8:**
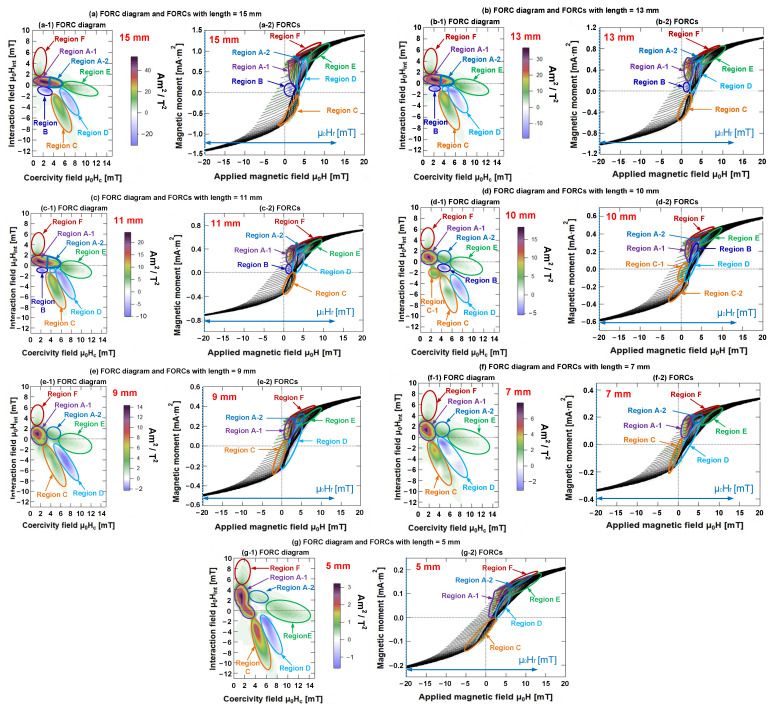
FORCs and FORC diagrams of Wiegand wires at varying lengths. (**a**) Length = 15 mm; (**b**) length = 13 mm; (**c**) length = 11 mm; (**d**) length = 10 mm; (**e**) length = 9 mm; (**f**) length = 7 mm; and (**g**) length = 5 mm.

As discussed earlier, the specific measurement method of FORCs was employed, and the applied magnetic field intensity ranged from µ_0_H_r_ = –500 mT to µ_0_H_sat_ = 500 mT. Each FORC curve was tracked by adjusting the magnetization of the Wiegand wire by varying the reversal field µ_0_H_r_ in steps of 0.5 mT. The measurement results of the FORCs of the Wiegand wire at varying lengths from µ_0_H = −75 to 75 mT are presented in Figure 8, and the FORC diagrams were derived from the FORCs.

In this work, we clearly elucidated the FORC distribution of different coercivity components of the Wiegand wire. When the length of the Wiegand wire was changed, the interactions among the different internal magnetic components, coercivity components, and FORC distribution would also change.

According to the FORC distribution trend and polarity in the FORC diagrams, we classified the distribution in each FORC diagram into several small regions, as illustrated in Figure 8(a-1–g-1). Moreover, their corresponding FORCs and the regions on FORCs are depicted in Figure 8(a-2,b-2,c-2,d-2,e-2,f-2,g-2), respectively.

We can obtain information on the magnetization process and magnetic interaction inside the sample from the FORC diagram [28]. The FORC distribution principally emphasizes an irreversible magnetization of the sample. When the lengths of the Wiegand wire were 15, 13, and 11 mm, the FORC distributions were divided into seven regions, as shown in Figure 8(a-1–c-1), respectively. Region A (regions A-1 and A-2) represents the irreversible magnetization of the sample, which is horizontally distributed along the µ_0_H_c_ axis. With the decrease in length, the distribution of region A tends to shorten. The lengths of the Wiegand wire being 10 and 9 mm, the FORC distribution was divided into eight and six regions, respectively, as shown in Figure 8(d-1,e-1). Only region A-1 represents the irreversible magnetization of the sample, which is distributed into an approximately circular region. The lengths of the Wiegand wire were 7 and 5 mm, and the FORC distribution was divided into six regions, as shown in Figure 8(f-1,g-1). Regions A-1 and C represent the irreversible magnetization of the sample, which is distributed in one direction between the µ_0_H_int_ and µ_0_H_C_ axes.

The FORC diagrams of the Wiegand wires with varying lengths were analyzed and compared. It was found that positive and negative distribution regions existed in each FORC diagram.

## 4. Discussion

### 4.1. Relationship between Coercivity and FORC Distribution

**Figure 9:**
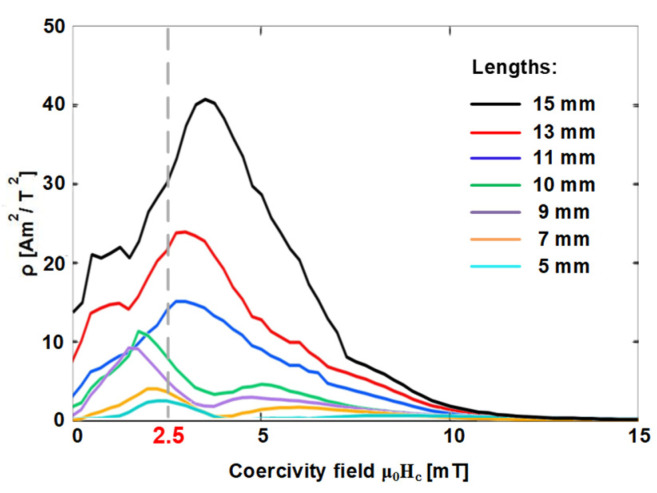
Intensities of FORC distribution under the interaction field μ_0_H_int_ = 0 mT of Wiegand wires at varying lengths and field intensities.

In the preliminary sections of this study, we showed that the coercivity of a given Wiegand wire remained almost constant at approximately 2.5 mT, and it did not vary with its length. Figure 9 shows the intensity of the FORC distribution of the Wiegand wires under the interaction field μ_0_H_int_ = 0 mT. As shown, the intensity of the FORC distribution decreased with the decrease in the Wiegand wire length. The maximum value of the FORC distribution of varying lengths appeared at the different field intensity from the coercive field μ_0_H_c_ = 2.5 mT. This difference is attributed to the fact that the FORC distribution represents the irreversible changes of magnetization.

### 4.2. Three-Layer Magnetic Structure and Its Magnetization Reversal

The FORC diagrams of all seven Wiegand wires were analyzed and compared. The findings indicated the gradual variation of the FORC distribution of these FORC diagrams, and the features of the FORC distribution of Wiegand wires with the lengths of 15, 10, and 5 mm were the most representative. Therefore, we considered three FORC diagrams of Wiegand wire with lengths of 15, 10, and 5 mm for analysis and a later discussion of the magnetic structure and interactions of Wiegand wires at varying lengths.

First, we regarded the FORC diagram and FORCs of the Wiegand wire with a length of 15 mm to analyze the attributes of each region in the FORC diagram. As illustrated in Figure 8a,b, Region A was distributed along the μ_0_H_c_-axis. For the analysis, we classified Region A into two regions, namely, A-1 and A-2. From the FORCs, several Barkhausen jumps were observed in region A-1; μ_0_H_c_ ranged from 0.25 to 3.50 mT, and μ_0_H_int_ ranged from −0.25 to 1.50 mT. The intensity of the interaction field was relatively low. Thus, Region A-1 can be considered as the soft layer of the Wiegand wire with small positive interaction.

Region A-2 was situated at the top of the Barkhausen jump on the FORCs, where μ_0_H_c_ ranged from 3.50 to 5.75 mT, and μ_0_H_int_ ranged from −0.25 to 1.50 mT. Region A-2 can be considered as an intermediate layer of the Wiegand wire with small positive interaction, whereas Region B was situated at the bottom of the Barkhausen jump (in addition to certain additional jumps) on the FORCs. In particular, μ_0_H_c_ ranged from 1.25 to 3.75 mT, whereas μ_0_H_int_ ranged from −0.25 to −1.75 mT. Therefore, Region B can be considered as the soft layer of Wiegand wire with small negative interaction. Regions A and B will evolve if the length is reduced.

Region C was primarily distributed at the starting position of the Barkhausen jump on multiple FORCs with μ_0_H_c_ ranging from 3.75 to 7.25 mT, and μ_0_H_int_ ranging from −1.75 to −8.50 mT. Moreover, Region D was mainly distributed at the end of the Barkhausen jump on various FORCs, with μ_0_H_c_ in the range of 5.0–8.75 mT and μ_0_H_int_ in the range of −1 to −5.75 mT. Thus, the formation of Regions C and D was attributed to the interaction between various layers inside the Wiegand wire with evident interactions. Furthermore, Region E was distributed away from the Barkhausen jump on multiple FORCs, where μ_0_H_c_ ranged from 6.0 to 12.25 mT, and μ_0_H_int_ ranged from −2 to 1 mT. In principle, we considered Region E as the hard core of the Wiegand wire with small positive interaction. Additionally, Region F was distributed near Region A on various FORCs with μ_0_H_c_ ranging from 0.25 to 2.75 mT, but μ_0_H_int_ extended from 1.50 to 7.0 mT. Therefore, the formation of Region F was potentially caused by the evident interaction between various layers inside the Wiegand wire.

Following the same method, we analyzed the attributes of each region in the FORC diagram of Wiegand wires with lengths equal to 10 and 5 mm, respectively. The attribute statistics of the various regions in each FORC diagram are listed in Table 1.

### 4.3. Positive and Negative Distributions in FORC Diagrams

As observed, each FORC diagram contained one or two blue regions, thus, implying that the FORC distribution was negative (ρ(H, H_r_) < 0). Conversely, the other remaining regions indicated that the FORC distribution was positive (ρ(H, H_r_) > 0). The origin of the negative regions in the FORC diagrams was reported earlier [11,28,29]. According to Equation (1) of the FORC distribution, we can determine that the positive or negative FORC distributions are related to the variations in the slope of various FORCs at the point of an applied magnetic field μ_0_H. The evidence of this conclusion was found in the FORC diagram and the corresponding FORCs.

We can define $k=\partial M(H,H_{r})/\partial H$ as the slope of various FORCs with distinct reversal fields H_r_ for magnetic field intensities H. As the reversal field H_r_ decreased, the slope of various FORCs $k=\partial M(H,H_{r})/\partial H$ increased with the applied magnetic field H; $\Delta H_{r}$ was negative, and $\Delta \left[\partial M(H,H_{r})/\partial H\right]$ was positive. Consequently, the FORC distribution ρ(H, H_r_) was positive. If H_r_ decreased along with the slope of various FORCs $k=\partial M(H,H_{r})/\partial H$ at the applied magnetic field H, both $\Delta H_{r}$ and $\Delta \left[\partial M(H,H_{r})/\partial H\right]$ were negative along with the FORC distribution ρ(H, H_r_).

**Figure 10:**
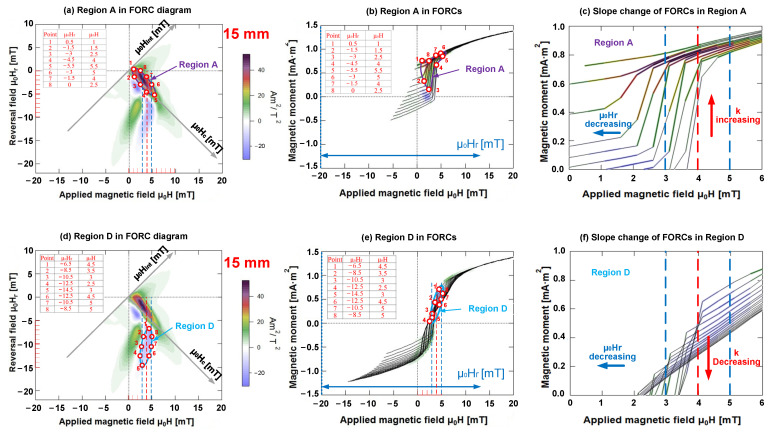
Comparison of positive and negative distributions in FORC diagram in same applied magnetic field. (**a**) Region A in FORC diagram; (**b**) Region A in several FORCs; (**c**) slope change of FORCs in region A; (**d**) Region D in FORC diagram; (**e**) Region D in several FORCs; (**f**) slope change of FORCs in region D.

Let us consider the FORC diagram of the Wiegand wire (length = 15 mm) as an example, which is illustrated in Figure 10a,d. The FORC distribution was positive if the applied magnetic field μ_0_H ranged from 3 to 5 mT, and the reversal field μ_0_H_r_ varied between 0 and −5.5 mT; however, it was negative if the reversal field μ_0_H_r_ ranged from −6.5 to −14.5 mT. The slopes of various FORCs were compared at the midpoint of 3–5 mT, i.e., the applied magnetic field μ_0_H = 4 mT, as illustrated in Figure 10b,e. As observed, as the reversal field μ_0_H_r_ decreased, the slope of the FORC curves corresponding to the region with positive FORC distribution increased, whereas the slope of the FORC curves corresponding to the region with negative FORC distribution diminished, as illustrated in Figure 10c,f. These explanations are consistent with the mathematical definition of the FORC distribution.

### 4.4. Relationship between Magnetization Reversal Direction and FORC Distribution

**Figure 11:**
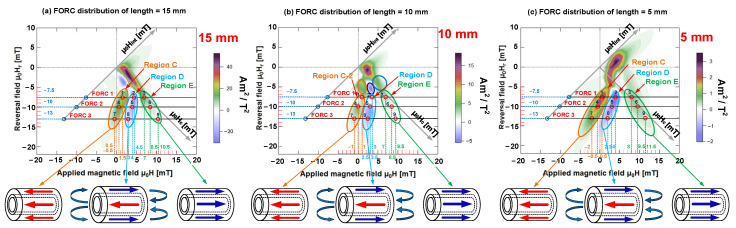
Three FORC diagrams with lengths of 15, 10, and 5 mm, and three FORCs with distinct reversal fields μ_0_H_r_ of −7.5, −10, and −13 mT. Relationships between the magnetization reversal direction and the positive and negative distributions of FORC diagrams. (**a**) The FORC distribution with a Wiegand wire of length 15 mm. (**b**) The FORC distribution with a Wiegand wire of length 10 mm. (**c**) The FORC distribution with a Wiegand wire of length 5 mm.

In addition, we considered three FORC diagrams of Wiegand wire with lengths of 15, 10, and 5 mm as an example. In each FORC diagram, we analyzed and discussed the relationship between three distinct regions and FORCs based on three FORCs (FORC 1, FORC 2, and FORC 3) with reversal fields μ_0_H_r_ set as −7.5, −10, and −13 mT, respectively. Specifically, these FORCs simultaneously passed through the regions C, D, and E in the FORC diagrams. We considered three distinct points on each FORC curve that were located in regions C, D, and E in every FORC diagram. Thus, nine points were selected in each FORC diagram, as illustrated in Figure 11.

**Figure 12:**
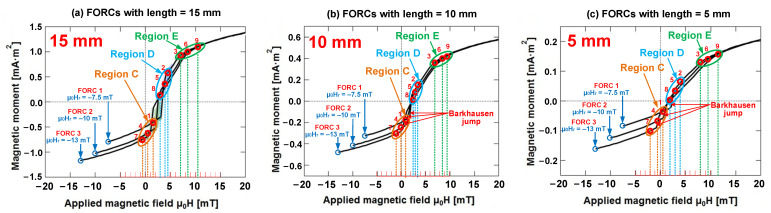
Three FORCs of each Wiegand wire with varying lengths if the reversal fields are −7.5 mT, −10 mT, and −13 mT. (**a**) Three FORCs with Wiegand wire lengths equal to 15 mm. (**b**) Three FORCs with Wiegand wire lengths equal to 10 mm. (**c**) Three FORCs with Wiegand wire lengths equal to 5 mm.

As illustrated in Figure 12, three FORCs of each Wiegand wire with varying lengths for the reversal fields were −7.5, −10, and −13 mT, respectively. Upon combining the FORC curve and the corresponding FORC diagram, we determined that three typical points in Region C (points 1, 4, and 7) were respectively distributed in positions located before the Barkhausen jumps on the three distinct FORCs. Additionally, three typical points in Region D (points 2, 5, and 8) were correspondingly distributed at positions after the Barkhausen jumps on the three distinct FORCs, and three typical points in Region E (points 3, 6, and 9) were respectively distributed in positions situated farther from the Barkhausen jumps on three distinct FORCs. In these FORCs, at the same applied magnetic field intensity, the switching field of the Barkhausen jump observably decreased with the decrease in Wiegand wire length. This finding is consistent with the conclusion on minor loops, as mentioned in Section 3.1.

If the minor loop is compared with the FORC curve, certain regions on the minor loop correspond to the typical points on the FORC curve, as illustrated in Figure 2c,d. In particular, the distinct FORC distribution regions in each FORC diagram indicated the presence of several unique field components of magnetization reversal in the Wiegand wire. If there was no interaction between these components, they usually formed a peak or ridges along the zero-bias axis (μ_0_H_int_ = 0) [30]. These unique field components were distributed along the H_int_-axis of the FORC diagram. The interaction between the components caused them to deviate from the zero-bias axis and/or result in the formation of ridges [28]. This signified the presence of interactions between the soft layer and hard core of the Wiegand wire [11].

As illustrated in Figure 11, the magnetization reversal directions of the soft layer and hard core remained unchanged, the FORC distribution was positive, and for the opposite direction, the FORC distribution was negative, owing to the magnetostatic coupling. This resulted from the interaction between the soft layers and hard core in the Wiegand wire, which was consistent with the previous research conclusions.

## 5. Conclusions

In this study, the magnetization properties of the Wiegand wire were examined based on minor loops, FORCs, and FORC diagrams. The magnetic structure of the Wiegand wire and its internal interaction were clarified successfully based on the detailed analysis of the prominent features in the FORCs and FORC diagrams.

(1) Regardless of the length of the Wiegand wire, its coercive field remained constant at approximately 2.5 mT because the measured coercivity in a major loop was the weighted coercivity of the entire ensemble of magnetic particles that constitute a magnetic material, and the major loops were symmetric magnetization curves of the whole sample.
(2) The switching field yielding the Barkhausen jump varied with the Wiegand wire length, which was attributed to the demagnetizing field depending on the wire length. The soft layer of short-length wires was autonomously reversed by a large demagnetizing field.
(3) In addition to the soft layer and hard core, an intermediate layer was observed in the magnetic structure of the Wiegand wire. Specifically, a transition region existed between the soft layer and hard core, referred to as the intermediate layer. The coercivity was considered gradually vary along the radial direction of the Wiegand wire.
(4) Based on the definition of the FORC distribution function and the analysis of the variation trends of the FORCs, we determined that the positive and negative regions in the FORC diagrams were related to the variations of the reversal field and the slope of the FORCs. Moreover, owing to the interaction between various layers inside the Wiegand wire, the magnetization reversal direction of the soft layer and hard core remained unchanged, and the FORC distribution was positive; when the direction was opposite, the FORC distribution was negative. Accordingly, both the positive and negative distributions in the FORC diagram including the physical interaction mechanism were defined mathematically.

Therefore, the FORCs and FORC diagram provided suitable evidence for the definition of the magnetic structure of the Wiegand wire, which cannot be evaluated by the conventional magnetization hysteresis curve, as they are indispensable for characterizing the interactions and coercivity distributions of the Wiegand wire.

## Figures and Tables

**Table 1:** Attribute statistics of various areas in each FORC diagram.

Areas	Length = 15 mm			Length = 10 mm			Length = 5 mm		
	µ_0_H_c_ (mT)	µ_0_H_int_ (mT)	Attribute	µ_0_H_c_ (mT)	µ_0_H_int_ (mT)	Attribute	µ_0_H_c_ (mT)	µ_0_H_int_ (mT)	Attribute
**Region A-1**	0.25–3.50	−0.25 to 1.50	Soft layer	0.25–3.50	−0.25 to 2.0	Soft layer	0.25–3.75	−1.75 to 4.75	Soft layer
**Region A-2**	3.50–5.75	−0.25 to 1.50	Intermediate layer	3.50–5.75	−0.25 to 2.0	Intermediate layer	3.0–6.25	1.75 to 3.75	Intermediate layer
**Region B**	1.25–3.75	−0.25 to −1.75	Soft layer	3.25–5.75	−0.50 to −2.0	Intermediate layer	\	\	\
**Region C-1**	\	\	\	1.75–3.75	−1.50 to −3.0	Soft layer	\	\	\
**Region C**	3.75–7.25	−1.75 to −8.50	Interaction	3.75–6.75	−2.50 to 9.25	Interaction	3.25–6.75	−1.75 to −11.25	Interaction
**Region D**	5.0–8.75	−1.0 to −5.75	Interaction	6.0–9.50	−2.50 to 7.75	Interaction	5.0–9.0	−1.0 to −8.25	Interaction
**Region E**	6.0–12.25	−2.0 to 1.0	Hard core	5.75–12.0	−2.0 to 0.75	Hard core	6.0–14.0	−2.50 to 2.0	Hard core
**Region F**	0.25–2.75	1.50 to 7.0	Interaction	0.25–2.75	2.0 to 6.25	Interaction	0.25–3.0	5.0 to 10.0	Interaction

## Data Availability

Not applicable.

## References

[1] Wiegand J.R., Velinsky M. (1974). Bistable Magnetic Device. U.S. Patent.

[2] Takemura Y., Fujiyama N., Takebuchi A., Yamada T. (2017). Battery-less hall sensor operated by energy harvesting from a single Wiegand pulse. IEEE Trans. Magn..

[3] Takahashi K., Takebuchi A., Yamada T., Takemura Y. (2018). Power supply for medical implants by Wiegand pulse generated from a magnetic wire. J. Magn. Soc. Jpn..

[4] Matsushita A., Abe S. (1979). Induced pulse voltage in twisted ferromagnetic wire. IEEJ Trans. A.

[5] Matsushita A., Takemura Y. (2000). Power generating device using compound magnetic wire. J. Appl. Phys..

[6] Takemura Y., Matsushita Y. (2001). Frequency dependence of output voltage generated from bundled compound wires. IEEE Trans. Magn..

[7] Yang C., Sakai T., Yamada T., Song Z., Takemura Y. (2020). Improvement of pulse voltage generated by Wiegand sensor through magnetic-flux guidance. Sensors.

[8] Serizawa R., Yamada T., Masuda S., Abe S., Kohno S., Kaneko F., Takemura Y. Energy harvesting derived from magnetization reversal in FeCoV wire. Proceedings of the IEEE Sensors.

[9] Zhao Y.-H. (1996). Wiegand Magnetic Sensors with Zero Power Dissipation. Instrum. Tech. Sens..

[10] Chang C.-C., Chang J.-Y. (2020). Novel Wiegand-effect based energy harvesting device for linear magnetic positioning system. Microsyst. Technol..

[11] Yang C., Kita Y., Song Z., Takemura Y. (2021). Magnetic reversal in Wiegand wires evaluated by first-order reversal curves. Materials.

[12] Takahashi K., Yamada T., Takemura Y. (2019). Circuit parameters of a receiver coil using a Wiegand sensor for wireless power transmission. Sensors.

[13] Sun X., Yamada T., Takemura Y. (2019). Output characteristics and circuit modeling of Wiegand sensor. Sensors.

[14] 8600 Series Vibrating Sample Magnetometry (VSM). http://www.linkphysics.com/product/165-cn.html?dynType=8&dynId=article.384839,306061,306064,384797&dynIgnoredWidget=article&bd_vid=8070893545908112954.

[15] Nakamura T., Tanaka H., Horiuchi T., Yamada T., Takemura Y. (2021). Surface magnetization reversal of Wiegand wire measured by the Magneto-Optical Kerr Effect. Materials.

[16] García J., Manterola A.M., Méndez M., Fernández-Roldán J.A., Vega V., González S., Prida V.M. (2021). Magnetization reversal process and magnetostatic interactions in Fe_56_Co_44_/SiO_2_/Fe_3_O_4_ core/shell ferromagnetic nanowires with non-magnetic interlayer. Nanomaterials.

[17] Pike C.R., Roberts A.P., Verosub K.L. (1999). Characterizing interactions in fine magnetic particle systems using first order reversal curves. J. Appl. Phys..

[18] Dodrill B., Lindemuth J., Radu C., Reichard H. (2015). White paper: High-temperature FORC study of single- and multi-phase permanent magnets. MRS Bull..

[19] Dodrill B., Ohodnicki P., Leary A., McHenry M. (2016). High-temperature first-order-reversal-curve (FORC) study of magnetic nanoparticle based nanocomposite materials. MRS Adv..

[20] Pike C.R., Roberts A.P., Dekkers M.J., Verosub K.L. (2001). An investigation of multi-domain hysteresis mechanisms using FORC diagrams. Phys. Earth Planet. Inter..

[21] Roberts A.P., Pike C.R., Verosub K.L. (2000). First-order reversal curve diagrams: A new tool for characterizing the magnetic properties of natural samples. J. Geophys. Res..

[22] Muster K.S., Heindla R. (2020). Determination of demagnetizing factors using first-order reversal curves and ferromagnetic resonance. AIP Adv..

[23] Mohammad R.Z.K., Bethanie S. (2021). Exploring effects of magnetic nanowire arrangements and imperfections on first-order reversal curve diagrams. IEEE Trans. Magn..

[24] Shi Z.-X., Zhou D.-Y., Li S.-D., Xu J., Uwe S. (2021). First-order reversal curve diagram and its application in investigation of polarization switching behavior of HfO2-based ferroelectric thin films. Acta Phys. Sin..

[25] Takemura Y., Aoki T., Tanaka H., Yamada T., Abe S., Kohno S., Nakamura H. (2006). Control of demagnetizing field and magnetostatic coupling in FeCoV wires for zero-speed sensor. IEEE Trans. Magn..

[26] Harrison R.J., Feinberg J.M. (2008). FORCinel: An improved algorithm for calculating first-order-reversal curve distributions Using Locally Weighted Regression Smoothing. Geochem. Geophys. Geosyst..

[27] Egli R. (2013). VARIFORC: An optimized protocol for calculating non-regular first-order reversal curve (FORC) diagrams. Glob. Planet. Chang..

[28] Muxworthy A.R., Roberts A.P., Gubbins D., Herrero Bervera E. (2007). First-order reversal cure (FORC) diagram. Encyclopedia of Geomagnetism and Paleomagnetism.

[29] Muxworthy A.R., King J.G., Heslop D. (2005). Assessing the ability of first-order reversal curve (FORC) diagrams to unravel complex magnetic signals. J. Geophys. Res..

[30] Acton G., Yin Q.Z., Verosub K.L., Jovane L., Roth A., Jacobsen B., Ebel D.S. (2007). Micromagnetic coercivity distributions and interactions in chondrules with implications for paleo intensities of the early solar system. J. Geophys. Res..

